# An investigation into IgE-facilitated allergen recognition and presentation by human dendritic cells

**DOI:** 10.1186/1471-2172-14-54

**Published:** 2013-12-13

**Authors:** Inas K Sharquie, Abeer Al-Ghouleh, Patricia Fitton, Mike R Clark, Kathryn L Armour, Herb F Sewell, Farouk Shakib, Amir M Ghaemmaghami

**Affiliations:** 1 Faculty of Medicine and Health Sciences, Division of Immunology, University of Nottingham, Queen’s Medical Centre, Nottingham, UK; 2 Immunology Division, Department of Pathology, University of Cambridge, Cambridge, UK; 3 AKS current address: College of Medicine, Baghdad University, Baghdad, Iraq

**Keywords:** Allergen, Dendritic cells, Der p 1, IgG, IgE

## Abstract

**Background:**

Allergen recognition by dendritic cells (DCs) is a key event in the allergic cascade leading to production of IgE antibodies. C-type lectins, such as the mannose receptor and DC-SIGN, were recently shown to play an important role in the uptake of the house dust mite glycoallergen Der p 1 by DCs. In addition to mannose receptor (MR) and DC-SIGN the high and low affinity IgE receptors, namely FcϵRI and FcϵRII (CD23), respectively, have been shown to be involved in allergen uptake and presentation by DCs.

**Objectives:**

This study aims at understanding the extent to which IgE- and IgG-facilitated Der p 1 uptake by DCs influence T cell polarisation and in particular potential bias in favour of Th2. We have addressed this issue by using two chimaeric monoclonal antibodies produced in our laboratory and directed against a previously defined epitope on Der p 1, namely human IgE 2C7 and IgG1 2C7.

**Results:**

Flow cytometry was used to establish the expression patterns of IgE (FcϵRI and FcϵRII) and IgG (FcγRI) receptors in relation to MR on DCs. The impact of FcϵRI, FcϵRII, FcγRI and mannose receptor mediated allergen uptake on Th1/Th2 cell differentiation was investigated using DC/T cell co-culture experiments. Myeloid DCs showed high levels of FcϵRI and FcγRI expression, but low levels of CD23 and MR, and this has therefore enabled us to assess the role of IgE and IgG-facilitated allergen presentation in T cell polarisation with minimal interference by CD23 and MR. Our data demonstrate that DCs that have taken up Der p 1 via surface IgE support a Th2 response. However, no such effect was demonstrable via surface IgG.

**Conclusions:**

IgE bound to its high affinity receptor plays an important role in Der p 1 uptake and processing by peripheral blood DCs and in Th2 polarisation of T cells.

## Background

Allergic diseases represent a major health problem affecting a large sector of the population [1,2]. Type I hypersensitivity, or allergy, is initiated by the recognition of an allergen by antigen presenting cells (mainly dendritic cells (DCs)), followed by a series of events that eventually result in IgE antibody production, mast cell sensitisation and triggering [3]. Allergen recognition by DCs represents the first step in allergic sensitisation and, therefore, is considered an attractive target for study since it might have an important role in determining subsequent downstream events of the allergic cascade [4].

Allergens, such as Der p 1, that cause these allergic reactions are generally innocuous proteins. Der p 1 is considered as the most immunodominant allergen of the house dust mite *Dermatophagoides pteronyssinus*[5]. It is a 25 kDa protein with cysteine protease activity. This protease activity is thought to be responsible for Der p 1 being a potent inducer of IgE synthesis, which is most probably mediated by the cleavage of regulatory molecules of IgE synthesis, such as CD23, CD25, CD40 and dendritic cell-specific intercellular adhesion molecule-3 (ICAM3)-grabbing non-integrin (DC-SIGN or DC209) [6].

DCs are professional antigen-presenting cells that occupy a central position at the interface of innate immunity and adaptive immune responses, through recognising foreign antigens, processing them and presenting them to T cell receptors via MHC molecules [7-9]. DCs use multiple pathways and cell-surface molecules for antigen capture and receptor-mediated endocytosis [10,11] which could influence T cell polarisation. 

In recent studies in our laboratory, it was shown that the C-type lectin receptors, mannose receptor (CD206 or MR) and DC-SIGN, play a significant role in Der p 1 uptake, internalisation and presentation. It has been shown that these receptors are characterised by the presence of carbohydrate recognition domains (CRD) that recognise sugar moieties on allergens [12-15]. The other two receptors thought to be involved in allergen uptake by DCs are IgE high and low affinity receptors, FcϵRI and FcϵRII (CD23) respectively. However, their precise roles in capturing allergen by DCs and subsequent presentation to T cells are not fully understood.

It has been previously suggested that IgE might play an important role in antigen uptake by DCs through IgE receptors [16]. It was also reported that the competence of antigen uptake by Langerhans cells increases significantly in the presence of IgE and its receptor [17]. In this context, numerous studies by Maurer and co-workers have emphasised the role of the high affinity IgE receptor on DCs in the internalisation of IgE-bound allergens and their presentation by the major histocompatibility complex (MHC) class II compartment in a Cathepsin S-dependent pathway [18-20]. The low affinity IgE receptor expressed by B cells was also shown to participate in antigen presentation and activation of T cells in a mouse model [21,22].

Together, these findings helped to formulate the hypothesis of our work, which is that IgE-mediated allergen presentation primes naïve T cells towards Th2 cell differentiation. The elucidation of this mechanism could clearly have therapeutic potential.

Previous work in our laboratory generated Der p 1-specific chimeric human IgE (IgE 2C7) and IgG (IgG1 2C7) antibodies consisting of mouse variable regions (Vκ and VH) joined to a human IgE or IgG1 constant regions, respectively [23,24]. It has been shown by using phage peptide libraries that the Der p 1 epitope targeted by 2C7 antibodies is Leu147-Gln160 [25], and this specificity is representative of a major component of the human IgE response to Der p 1 [23]. The availability of these two antibodies has therefore provided a unique opportunity to investigate the consequences of IgE- versus IgG-facilitated Der p 1 presentation by DCs.

## Results

### Time course expression of FcϵRI, FcϵRII (CD23), FcγRI and MR by monocyte derived DCs (Mo-DCs)

The kinetics of expression of FcϵRI, FcϵRII (CD23), FcγRI and mannose receptor were studied during the course of generating Mo-DCs, starting from day zero (monocyte) through to days 2, 4, 6 (immature) and 8 (mature).

**Figure 1:**
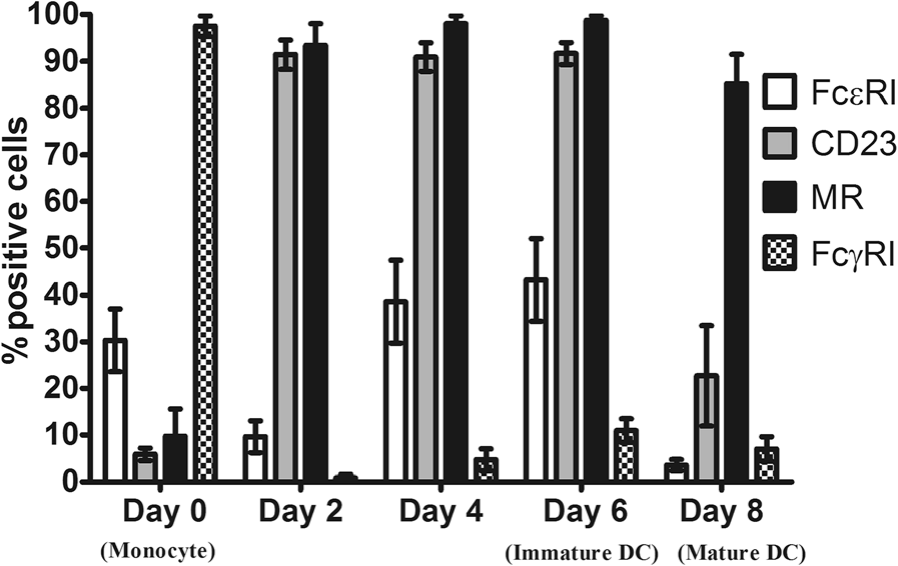
**Time-related FcϵRI, FcϵRII, FcγRI and MR expressions.** Receptor expression was detected during the course of generating DCs starting from day 0 (monocyte) through to days 2, 4, 6 (immature DC) and 8 (mature DC). The data presented show the average of six independent experiments for FcϵRI, CD23 and MR, and three independent experiments for FcγRI, all expressed as mean ± SEM.

FcϵRI was modestly expressed on monocytes at day zero, while FcϵRII and MR were less well expressed (Figure 1). On day two, FcϵRI expression decreased, but levels recovered on days 4 and 6. FcϵRII and MR expressions were at their maximum on days 2, 4 and 6 of culture (and day 8 in the case of MR). FcγRI expression was high to start with (day 0) then levels diminished in the course of generating the DCs.

### The specificity of IgE binding by Mo-DCs

**Figure 2:**
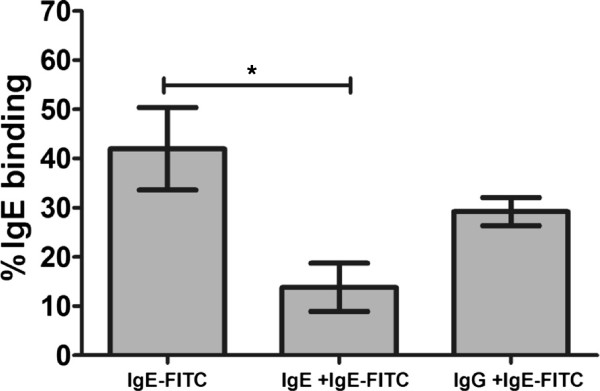
**Inhibition of IgE-FITC binding by Mo-DCs.** Immature dendritic cells (10^5^) were treated with IgE-FITC (7 μg/ml) plus blocking IgE or IgG antibodies (20 μg/ml) for 20 minutes at 4°C and then washed and fixed. Analysis was done by flow cytometry. The results show a significant decrease in IgE-FITC binding when mixed with unlabelled IgE, but not control IgG (*: p value ≤0.05). The data presented represent the average of three independent experiments expressed as mean ± SEM.

**Figure 3:**
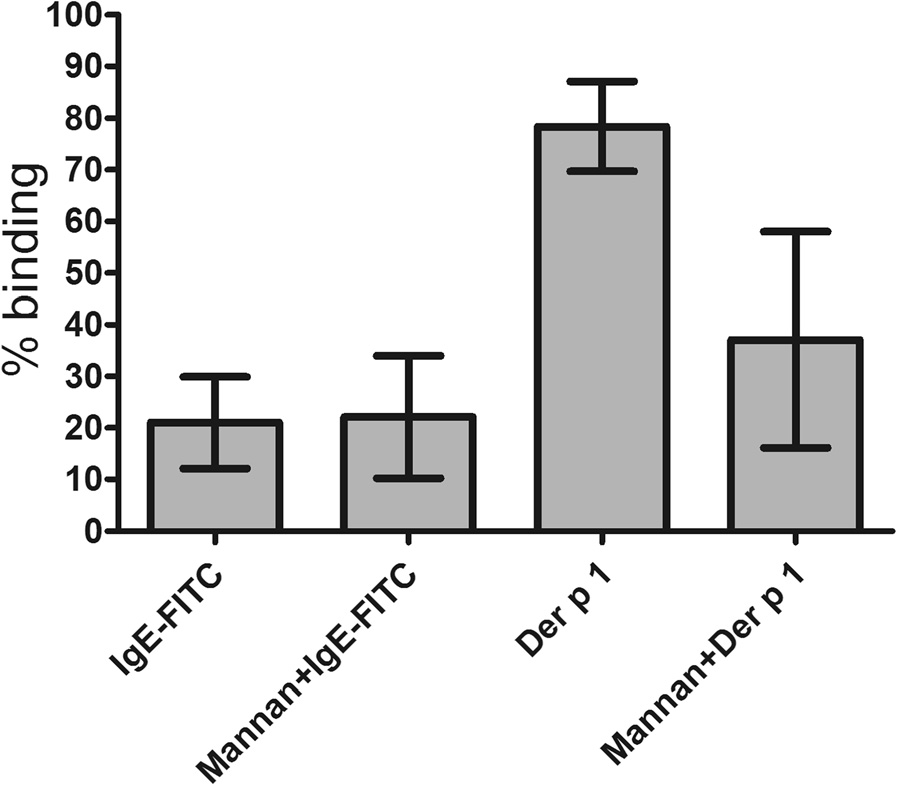
**Effect of mannan on the binding of IgE-FITC.** Immature Mo-DCs (10^5^) were treated with IgE-FITC (7 μg/ml) after pre-incubation with and without mannan (200 μg/ml) for 20 minutes at 4°C, and then washed and fixed. Analysis was done by flow cytometry. The data presented represent the average of two independent experiments expressed as mean ± SEM.

Immature DCs (day 6 of culture) were stained with IgE-FITC, in the presence or absence of unlabelled IgE or IgG, in order to determine IgE binding specificity. There was substantial inhibition of IgE-FITC binding when mixed with unlabelled IgE (p ≤0.05) but reduction in uptake in the presence of IgG was not significant (Figure 2). In further experiments, immature DCs (10^5^) were stained with IgE-FITC, in the presence or absence of mannan, in order to exclude IgE binding via the mannose receptor (i.e. through carbohydrates on IgE). Der p 1 uptake with and without mannan was used to show that mannan was active in the experiment. As shown in Figure 3, whilst able to block Der p 1 uptake, mannan had no effect on IgE binding to DCs.

### Sodium periodate oxidation of natural Der p 1 and the structural integrity of the deglycosylated allergen

**Figure 4:**
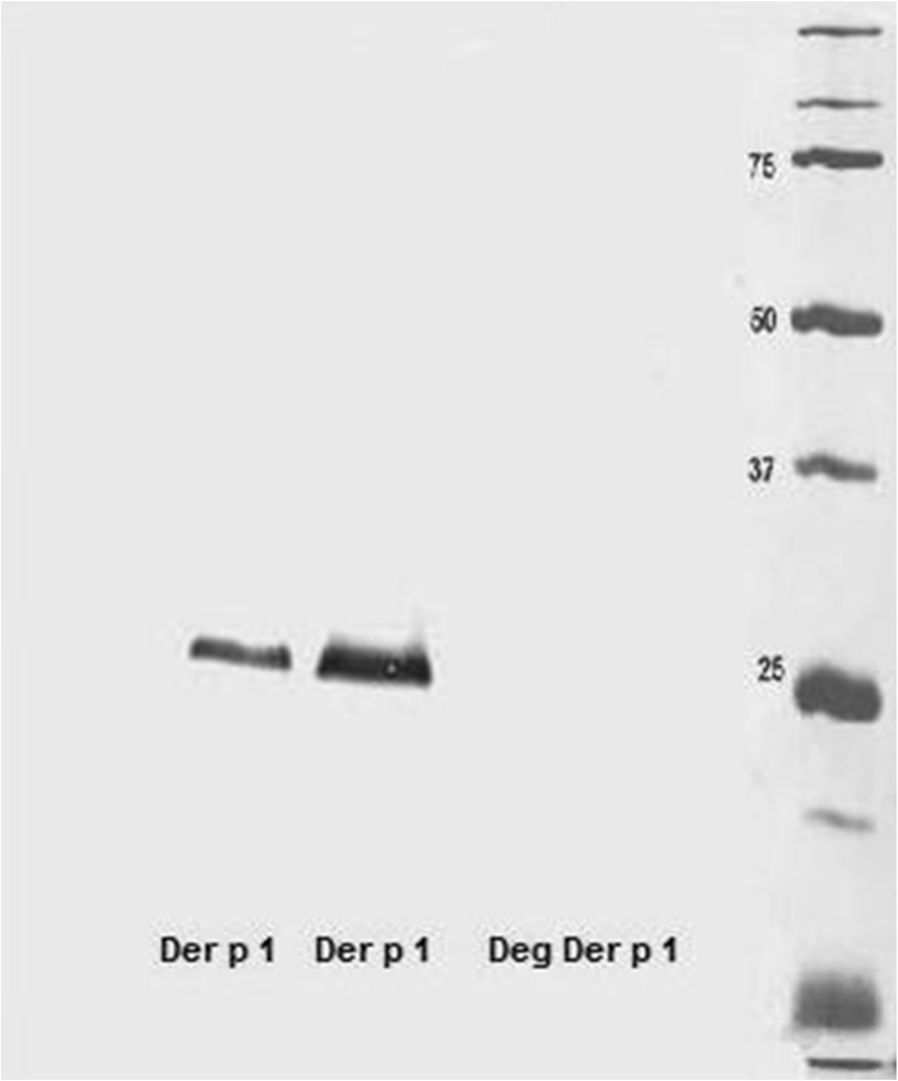
**The effect of sodium periodate on Der p 1.** Der p 1 was exposed to periodate for one hour and then immunoblotted alongside natural Der p 1 (as a control) against the anti-mannose sugar (GNA). The blot shows a very clear band with natural Der p 1, while no band can be seen with the deglycosylated Der p 1.

**Figure 5:**
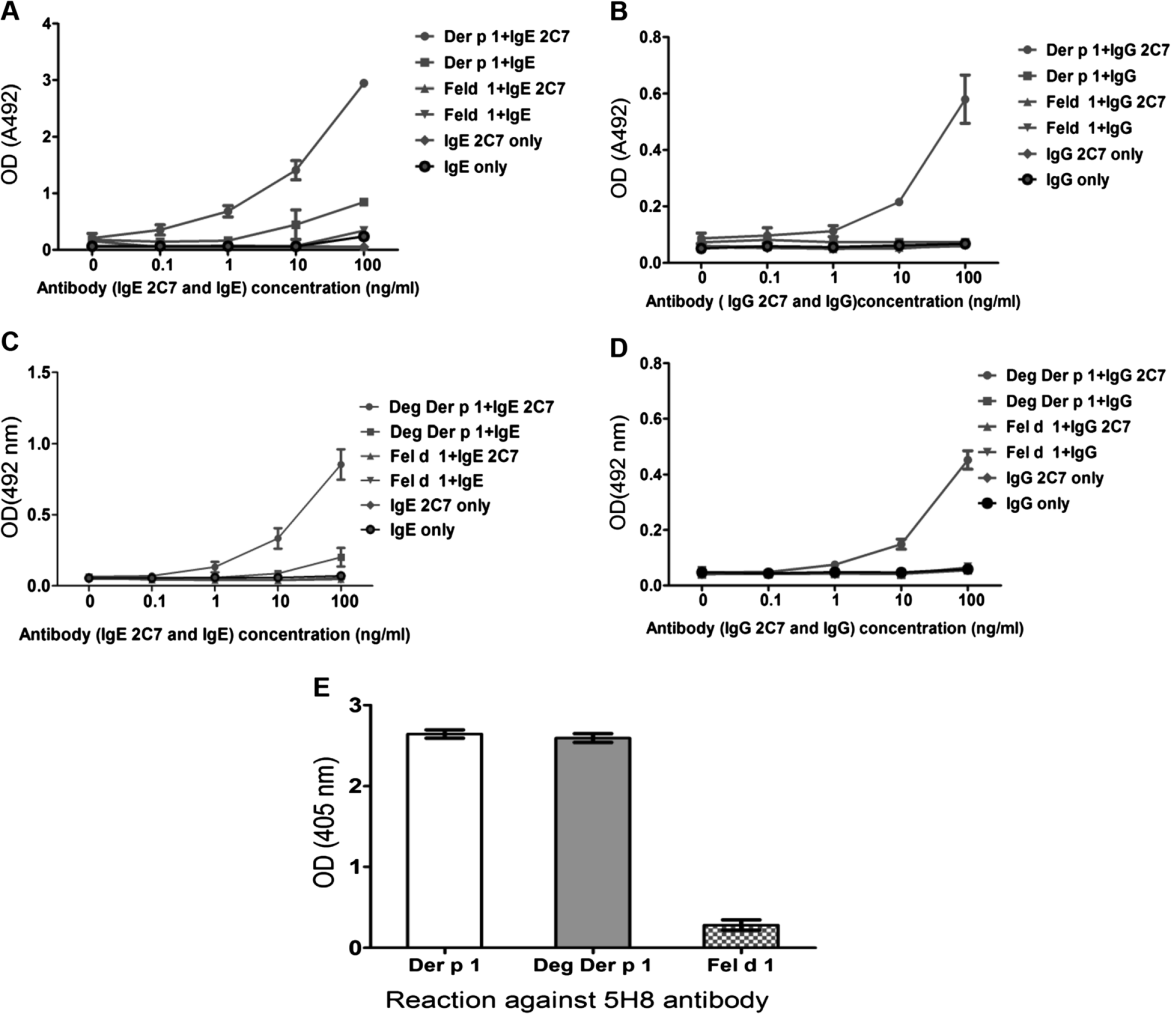
**ELISA results showing the reactivity of Der p 1 with monoclonal anti Der p 1 antibodies.** Wells were coated with 5 μg/ml of Der p 1 or a cat allergen (Fel d 1). IgE 2C7 **(A)** or IgG 2C7 **(B)** antibodies were used in different concentrations and binding detected using a goat anti-human κ chain HRP-conjugated secondary antibody. The data presented represent the average of two **(A)** or three **(B)** independent experiments expressed as mean ± SEM. In further experiments, wells were coated with 5 μg/ml of deglycosylated Der p 1 or Fel d 1. IgE 2C7 **(C)** or IgG 2C7 **(D)** antibodies were used in different concentrations and binding detected using a goat anti-human κ chain HRP-conjugated secondary antibody. The data presented represent the average of two independent experiments expressed as mean ± SEM. Analysis by ELISA of the binding of anti-Der p 1 5H8 antibody with periodate-treated Der p 1 **(E)** shows retention of Der p 1 protein structure. The data presented represent the average of three independent experiments expressed as mean ± SEM.

Der p 1 can be taken up efficiently by C-type lectin receptors (such as MR) on DCs via its sugar residues. To study the influence of the uptake of Der p 1 through immunoglobulins bound to DCs on T cell differentiation without MR engagement, we deglycosylated Der p 1 to exclude binding through MR. To confirm the deglycosylation of Der p 1, Western blotting of natural Der p 1 and deglycosylated Der p 1 against GNA (recognises terminal mannose 1–2, 1–3 and 1–6) was performed. Unlike natural Der p 1, no band was visible with the deglycosylated Der p 1 preparation (Figure 4). An ELISA was used to ascertain the structural integrity of the deglycosylated Der p 1. As shown in Figure 5, deglycosylated Der p 1 was just as good as natural Der p 1 in being recognised by the 2C7 and 5H8 antibodies

### Der p 1 reactivity of IgE and IgG 2C7 antibodies

An ELISA was used to ascertain the Der p 1 reactivity of the IgE and IgG 2C7 antibodies used in our experiments. Results clearly indicate that the 2C7 antibodies react specifically with Der p 1 and no such reactivity can be demonstrated with the control allergen (Fel d 1) or the isotypes control antibodies (Figure 5).

### IgE receptors play an important role in Der p 1 uptake by Mo-DCs

**Figure 6:**
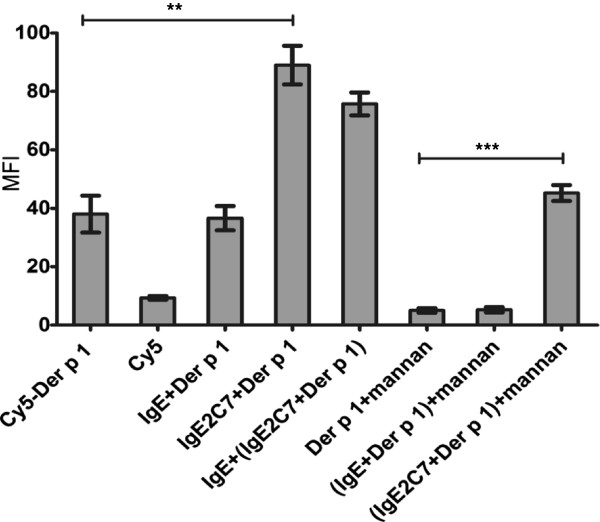
**Uptake of Der p 1 by Mo-DCs is mediated by IgE receptors.** The median fluorescent index for the uptake of Der p 1 (0.26 μg/ml Cy5 labelled), with and without human IgE (10 μg/ml), IgE 2C7 (1 μg/ml) or 200 μg/ml mannan, by immature Mo-DCs at 37˚. **: p ≤ 0.01; ***: p ≤ 0.001. The data presented represent the average of three independent experiments expressed as mean ± SEM.

To investigate the role of IgE in the uptake of Der p 1 by Mo-DCs, we incubated Cy5 labelled Der p 1, with and without IgE 2C7, with immature Mo-DCs at 37°C. The experiment showed that adding IgE 2C7 significantly increases Der p 1 uptake to a level over and above that mediated by MR (Figure 6). The average means of uptake of Der p 1-IgE 2C7 is significantly higher than that of Der p 1 alone (p-value = 0.0051, n = 3), and Der p 1-IgE 2C7 when the cells were treated with mannan (p-value = 0.0036, n = 3). When the cells were pre-treated with mannan, the means average of uptake of Der p 1-IgE 2C7 was significantly higher than that of Der p 1 (p-value = 0.0001, n = 3). In contrast, there was no noticeable difference between the uptake of Der p 1 only (without treating cells with mannan) and Der p 1-IgE 2C7 (when treating cells with mannan) (Figure 6), which clearly demonstrates that the MR- and specific IgE-mediated pathways make approximately equal contributions to Der p 1 uptake by Mo-DCs under these conditions.

### FcϵRI, FcϵRII (CD23), FcγRI and MR expressions by myeloid DCs

**Figure 7:**
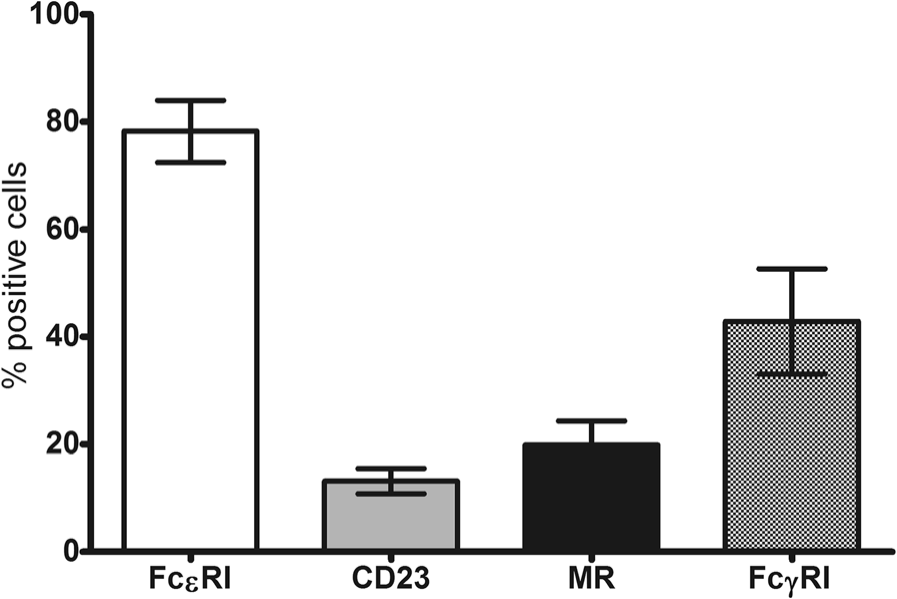
**FcϵRI, FcϵRII, FcγRI and MR expressions on myeloid DCs.** Peripheral blood myeloid DCs were stained with various monoclonal antibodies to detect FcϵRI, CD23, FcγRI and MR. The data presented are the average of four independent experiments expressed as mean ± SEM.

Myeloid DCs were stained with anti-FcϵRIα PE, anti-CD206 PC5, anti-CD23 ECD and anti-CD64 (FcγRI) FITC. As shown in Figure 7, FcϵRIα was expressed on high proportion of myeloid DCs, whereas CD23 and MR expressions were lower. FcγRI expression was detected on up to 43% of the myeloid DCs.

### Role of IgE in inducing Th1/Th2 cytokine production in myeloid DC-T cell co-cultures

Since myeloid (peripheral blood) DCs showed high level of FcϵRI and FcγRI (IgG high affinity receptor) expressions, with only minimal expressions of CD23 and MR, these cells provide an ideal opportunity to assess the role of IgE and IgG-facilitated allergen presentation in T cell polarisation with minimal or no interference by CD23 and MR.

Myeloid DCs that had been loaded with natural or deglycosylated Der p 1, both with and without IgE and IgG 2C7 antibodies, were co-cultured with autologous naïve T cells. IL-4, IL-5, IL-13 and IFN-γ secretions were then detected using a FlowCytomix kit after stimulation with PMA and Ionomycin.

**Figure 8:**
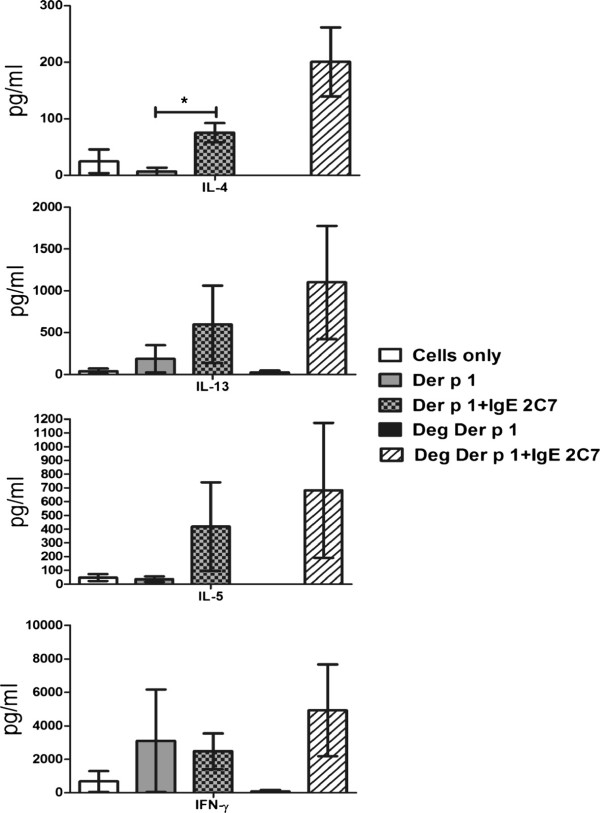
**Th1 and Th2 cytokine responses to stimulated myeloid DCs, demonstrating the effect of Der p 1 taken up via IgE or IgG 2C7 uptake on naïve T cells polarisation.** Myeloid DCs were pre-loaded with Der p 1 or deglycosylated Der p 1, both with and without IgE 2C7, prior to establishing DC-naïve T cell co-cultures. T cells were re-stimulated at day 10 with PMA (15 ng/ml) and Ionomycin (1ug/ml). The following conditions were used: cells only and Der p 1 or deglycosylated Der p 1, with and without IgE 2C7 (n = 3, except for deglycosylated Der p 1 only condition which was done twice). The results were statistically significant for IL-4 when myeloid DCs were pre-loaded with Der p 1 and IgE 2C7 (*:p ≤ 0.05).

**Figure 9:**
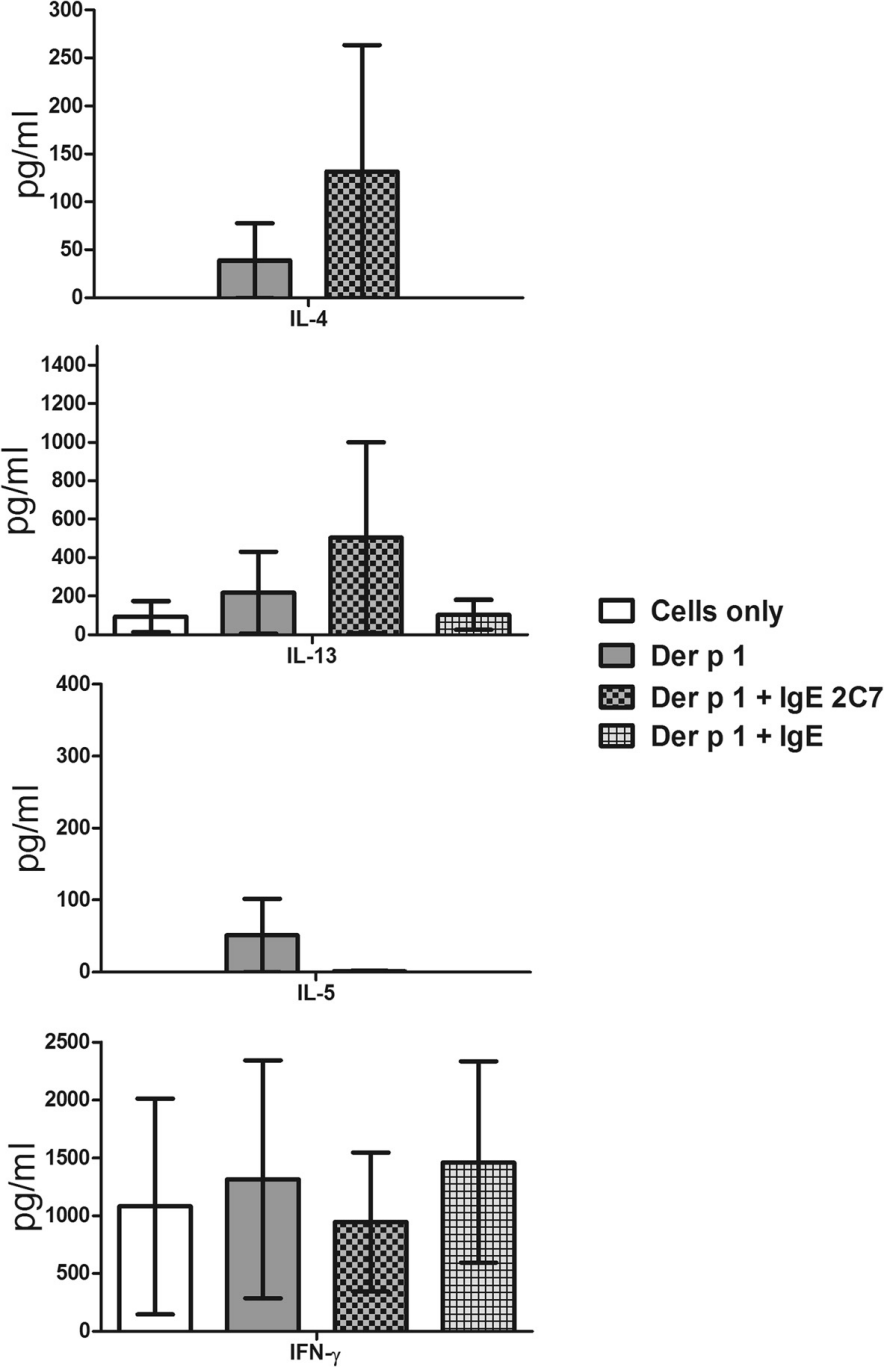
**Th1 and Th2 cytokine responses to stimulated myeloid DCs, demonstrating the effect of Der p 1 taken up via IgE 2C7, and using non-specific human IgE antibodies as controls, on naïve T cell polarisation.** Myeloid DCs were pre-loaded with Der p 1, with and without IgE 2C7 antibody, prior to establishing DC-naïve T cell co-cultures. T cells were re-stimulated at day 10 with PMA (15 ng/ml) and Ionomycin (1 ug/ml). The following conditions were used: cells only and Der p 1, with and without IgE 2C7 or IgE control (n =4). The data presented represent the average of four independent experiments expressed as mean ± SEM.

**Figure 10:**
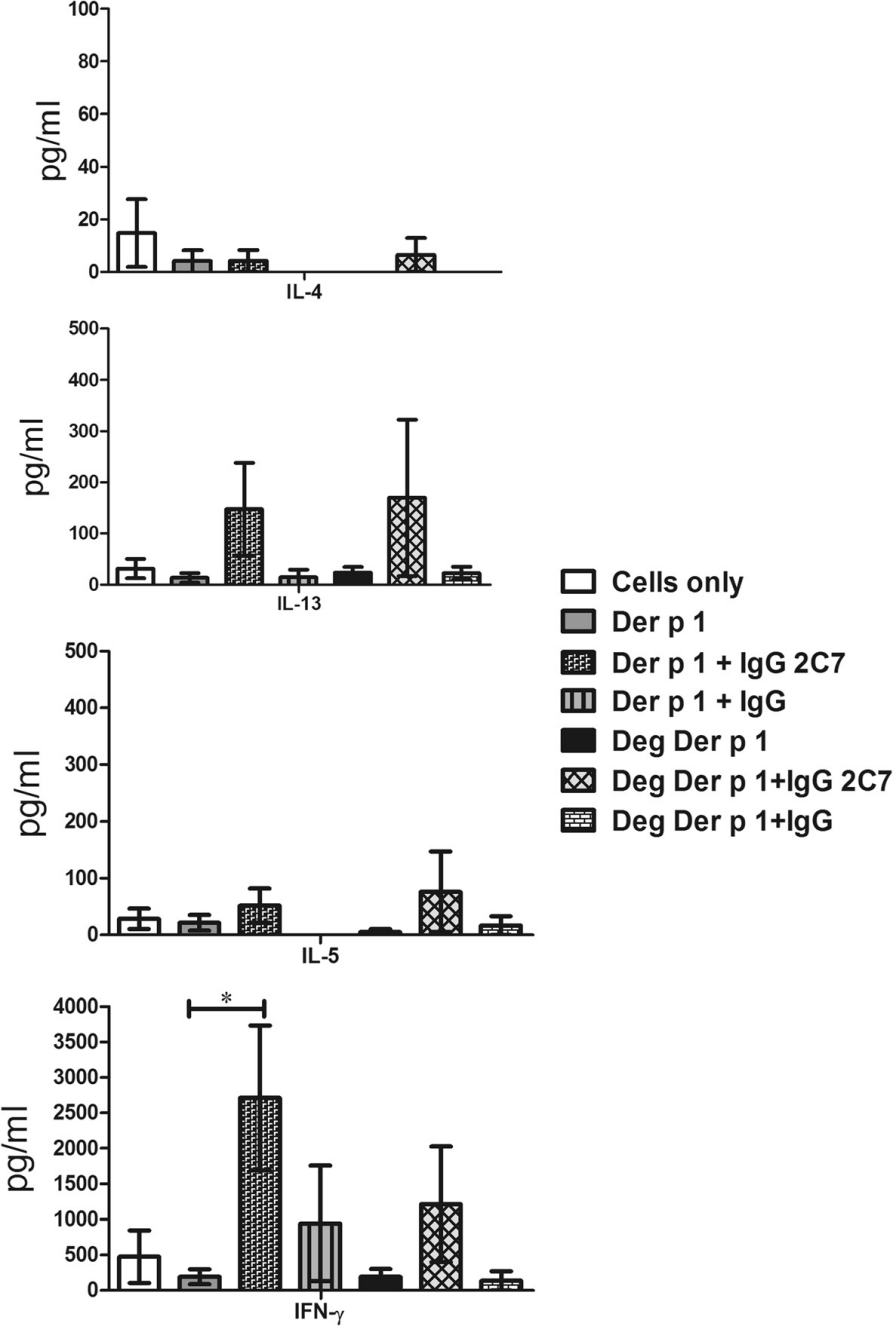
**Th1 and Th2 cytokine responses to stimulated myeloid DCs, demonstrating the effect of Der p 1 taken up via IgG 2C7, and using non-specific human IgG antibodies as controls, on naïve T cell polarisation.** Myeloid DCs were pre-loaded with Der p 1 or deglycosylated Der p 1, both with and without IgG 2C7 antibody, prior to establishing DC-naïve T cell co-cultures. T cells were re-stimulated at day 10 with PMA (15 ng/ml) and Ionomycin (1ug/ml). The following conditions were used: cells only, Der p 1 or deglycosylated Der p 1, with and without IgG 2C7 (n = 4 and n = 5, respectively), and Der p 1 or deglycosylated Der p 1, with IgG controls (n =3). The results were statistically significant for IFN-γ when myeloid DCs were pre-loaded with Der p 1 and IgG 2C7 (*:p ≤ 0.05).

The data demonstrated a high level of Th2 cytokine (IL-4, IL-5 and IL-13) secretion when the IgE 2C7 antibody was used with either Der p 1 or deglycosylated Der p 1, in comparison with using Der p 1 or deglycosylated Der p 1 alone (Figure 8). However, the results were only statistically significant for IL-4 (p ≤ 0.05) but not IL-5 or IL-13. IFN-γ production was high in all these conditions. As can be seen, deglycosylated Der p 1 gave rise to more cytokine production in the presence of IgE 2C7 than glycosylated Der p 1. This may have been due to deglycosylated Der p1 being bound more efficiently by IgE 2C7. Partial unmasking of the IgE 2C7 epitope could occur upon removal of sugar residues if these sterically limiting access by IgE 2C7. In further experiments, the IgE 2C7 antibody was compared to an isotype control (i.e. non-specific human IgE) and again higher concentrations of IL-4 and IL-13 cytokines were detected with IgE 2C7 (Figure 9). Importantly, no such effect was demonstrable with the IgG 2C7 antibody (Figure 10). Collectively, these data show the selectivity of IgE-facilitated Der p 1 uptake by DCs in terms of bias towards a Th2 phenotype. Whilst T cells in these co-cultures are most likely sources of the detected cytokines, it worth highlighting that measuring cytokines in the supernatant does not allow exact identification of the cellular source of the cytokines.

## Discussion

It is well accepted that FcϵRI is highly expressed on mast cells and basophils. However, a noticeable feature of FcϵRI on APCs is that its expression is highly variable, depending on the blood donor and the pathological situation [26]. We initially sought to monitor the kinetics of the expression of the high and low affinity IgE receptors (FcϵRI and FcϵRII [CD23], respectively), as well as the high affinity IgG receptor (FcγRI, CD64), in relation to MR during the generation of DCs from day zero (monocytes) to day 6 (immature DCs) and then finally day 8 (mature DCs). We successfully detected the expression of MR, FcϵRI, FcϵRII and FcγRI receptors on Mo-DCs as a basis for exploring labelled-IgE binding to Mo-DCs. The present study demonstrated a modest percentage (43%) of FcϵRI expression and high expression of CD23 (91%) and MR (98%) on immature Mo-DCs, in contrast to FcγRI, which proved to be expressed in very low levels.

IgE bound specifically to its receptors on immature Mo-DCs, as its binding was inhibited by IgE but not by IgG. The optimisation of IgE binding experiment revealed that the blocking effect of unlabelled IgE was only demonstrable when added at the same time as labelled IgE (i.e. in competition), and this could be due to the rapid turnover of FcϵRIα on the cell membrane.

DCs express the C-type lectin receptors MR and DC-SIGN, which have the ability to recognise mannose containing-carbohydrates on different antigens and pathogens. MR has specificity for proteins with mannose, fucose and N-acetyl glucosamine, while DC-SIGN recognises high mannose oligosaccharide structures [27,28]. Furthermore, it has been shown that IgE is rich in mannose and N-acetyl glucosamine oligosaccharides [29,30]. This, therefore, raises the possibility of IgE binding to DCs via C-type lectin receptors, particularly since previous studies have shown that IgE can be taken up by human alveolar macrophages via MR [31]. However, our data have shown that IgE binding by DCs is not mediated by C-type lectin receptors, as its binding was not inhibited by mannan, the natural ligand for MR and DC-SIGN.

The hypothesis that DCs could perform allergen capture and presentation via surface IgE is of great interest, since this could lead to further perpetuation of allergic tissue damage in atopic individuals where IgE circulates in very high concentrations. We were uniquely placed to test this hypothesis through the availability of IgE and IgG chimaeric human 2C7 antibodies directed against Der p 1, the major allergen of the HDM and an important cause of allergic asthma. This meant that we could study the role of IgE in allergen uptake on DCs and compare it to IgG, particularly since these antibodies have identical epitope specificity (i.e. same variable domains) and differ only in their constant region domains [24].

Having demonstrated IgE binding to its receptors (FcϵRI and FcϵRII) on DCs, we proceeded to investigate the role of IgE in Der p 1 uptake using a flow cytometry approach, whereby DCs were loaded with labelled Der p 1-IgE 2C7 complexes. Since Der p 1 can be internalised by MR and DC-SIGN [12,13,32], we used mannan, a polymer of mannose, to block MR and DC-SIGN in order to distinguish Der p 1 binding via IgE from that via C-type lectins. The results obtained show that Der p 1 uptake by DCs increases in the presence of IgE 2C7, but not when human IgE of irrelevant specificity was used as a control. Mannan significantly inhibited Der p 1 uptake by Mo-DCs when DCs were loaded with Der p 1 alone, but not when cells were loaded with Der p 1-IgE 2C7 complexes. This provides direct evidence that Der p 1 uptake by Mo-DCs is also mediated by a separate pathway (i.e. other than MR or DC-SIGN) involving IgE receptors.

The ultimate goal of this study was to explore the role of IgE in allergen uptake and its presentation by DCs via IgE receptors particularly FcϵRI, and to compare this with IgG. In line with previous studies [33,34], we found that myeloid DCs have very low expression of CD23, MR and DC-SIGN, but high expression of FcϵRI and FcγRI and are therefore ideally suited for our study. Because MR and DC-SIGN internalise Der p 1 via their carbohydrate binding specificity, we further used deglycosylated Der p 1 to explore the role of IgE in the uptake of Der p 1 without interference from any residual C-type lectins. The deglycosylated Der p 1 protein used reacted with the anti-Der p 1 5H8 antibody, as well as with 2C7 antibodies, thereby confirming its structural integrity.

It is well understood that the activation of CD4^+^ T cells leads their differentiation towards one of few developmental pathways such as Th1, Th2, Th17 or Treg depending on the concentration of different cytokines in the microenvironment amongst other factors [35,36]. Th2 cells and their cytokines (IL-4, IL-5 and IL-13) are abundant in allergic disease, and these cytokines play a central role in initiating allergic inflammation. Thus, we investigated the effect of IgE and IgG-facilitated Der p 1 uptake by peripheral blood myeloid DCs on downstream events at the T cell level.

The co-culture experiments showed that IgE facilitated Der p 1 uptake by myeloid DCs supported the differentiation of naïve T cells towards a Th2 cell phenotype. Given the low expression of CD23 on myeloid DCs this effect is most likely mediated via FcϵRI however we cannot rule out possible up-regulation of CD23 after sensitisation with IgE antibody hence a contribution from CD23 remains a possibility. The Th2 bias was evidenced by high levels of Th2 cytokine secretion, particularly IL-4, when the IgE 2C7 antibody was used in combination with natural Der p 1 or deglycosylated Der p 1. However, these findings did not reach statistical significance with two (i.e. IL-5 and IL-13) of the three Th2 cytokines tested most likely due to the low number of donors and wide inter donor variations obtained. Previous analysis of 55 crystal structures of known allergens (including Der p 1) has shown that dimerisation is a very common and essential feature of allergens [37]. We can therefore reasonably assume that although we were using a monoclonal IgE antibody (i.e. 2C7), which will not permit cross-linking by Der p 1 monomers, the biological effects observed in our DC-bound IgE/Der p 1 uptake experiments were due to IgE cross linking by Der p 1 dimers. However, given the importance of polyvalent cross-linking in efficient internalisation of FcϵRI-bound IgE [18] it is reasonable to expect that using a polyclonal anti Der p 1 IgE antibody could further enhance the observed biological effects such as Th2 cell polarisation. Interestingly, and as expected, we were unable to show a similar bias towards Th2 differentiation when using the IgG 2C7 antibody.

Our results are in keeping with a recent study by Sallmann and co-workers using a new transgenic mouse model, which suggested that FcϵRI expressed by DCs does indeed play a role in antigen uptake and the development of Th2 allergic tissue inflammation [38].

In conclusion, IgE could play an important role in Der p 1 uptake and processing by peripheral blood myeloid DCs and in the Th2 polarisation of T cells. This would clearly serve to perpetuate symptoms of allergy in atopic patients where IgE is already produced in large quantities. It is known that DCs prime Th2 cell polarisation after encountering allergens, but the particular mechanism by which DCs induce Th2, instead of Th1, development is not fully understood. The present work provides a potential mechanism by which myeloid DCs may support the development of Th2 responses and suggests a novel target for therapy.

## Conclusions

Whist C-type lectins play a key role in recognition and uptake of glyco-allergens including Der p 1, this study shows that IgE bound to its high affinity receptor plays an important role in the uptake and processing of Der p 1 by peripheral blood DCs as well as in Th2 polarisation of T cells. Such IgE mediated pathway of allergens uptake could be particularly important in individuals with high serum IgE levels who have already been sensitised to an allergen.

## Methods

### Der p 1 preparations

Der p 1 was purchased from Indoor Biotechnologies (Warminster, UK) and labelled with Cyanine 5 (Cy5) using a Cy5 labelling kit (GE Healthcare, Buckinghamshire, UK) according to the manufacturer’s instructions. The concentration of labelled allergens was determined by NanoDrop prior to use.

In order to deglycosylate natural Der p 1, sodium metaperiodate (Sigma-Aldrich, Irvine, UK) was used as previously described [39]. Briefly, Der p 1 was treated at a molar ratio of 5:1 with sodium periodate. Following incubation for one hour at room temperature in the dark, the oxidation process was stopped by adding 0.25 ml of ethylene glycol per ml of the reaction mixture. The deglycosylated samples were then dialysed at room temperature.

Deglycosylation was then confirmed using Western blot analysis. Briefly, using 12% Tris-Glycine precast gels (Invitrogen, Paisley, UK), gel electrophoresis was performed for 5 μg of natural and deglycosylated Der p 1 proteins, and these proteins were then transmitted to nitrocellulose membranes (GE Healthcare Life Sciences, Buckinghamshire, UK). Next, a DIG Glycan Differentiation kit (Roche Applied Science, Burgess Hill, UK) was used to determine the glycan content following the manufacturer’s instructions.

The structural integrity of the deglycosylated Der p 1 preparation was also tested against 2C7 and biotinylated 5H8 anti-Der p 1 antibodies, clone 5H8 C12, (Indoors Biotechnologies), using standard ELISA procedure in which the binding was detected using Extra Avidin alkaline phosphatase conjugate (Sigma-Aldrich, Irvine, UK).

### Generation of dendritic cells

Peripheral blood samples from non-atopic donors (obtained following local ethics committee approval and after obtaining informed consent) were used for separation of peripheral blood mononuclear cells (PBMCs) by density gradient centrifugation on Histopaque-1077 (Sigma-Aldrich, Irvine, UK). PBMCs were then incubated with mouse anti-human CD14 monoclonal antibody (mAb) conjugated to magnetic beads (Miltenyi Biotec, Bisley, UK). CD14^+^ monocytes were then collected using a magnetic cell separation system (Miltenyi Biotec, Bisley, UK).

Immature DCs were generated from CD14^+^ monocytes by culturing them with 50 ng/ml of Granulocyte-Macrophage colony stimulating factor (GM-CSF) and 250 IU/ml of IL-4 (both from R & D system, UK) in RPMI-1640 medium, supplemented with L-glutamine, penicillin and streptomycin (Sigma-Aldrich, Irvine, UK), and 10% FBS (Autogen Bioclear, Wiltshire, UK) (1 × 10^6^ cells/ml) in a 24-well flat bottom culture plate (Costar, High Wycombe, UK) at 37°C in 5% CO_2_ for six days as we have previously described [13,40,41]. The maturation of monocyte-derived DCs (Mo-DCs) was achieved by adding lipopolysaccharide (LPS) (200 ng/ml) (Sigma-Aldrich, Irvine, UK) to the immature DCs on day six and then incubating them for 48 hours at 37°C in a humidified atmosphere of 5% CO_2_.

In other experiments, where peripheral blood myeloid DCs (mDCs) (CD1c, BDCA-1) were used, these cells were separated from PBMCs by two magnetic separation steps using the CD1c (BDCA-1) + dendritic cell isolation kit from Miltenyi Biotech (Bisley, UK) and following the manufacturer’s guidelines. Briefly, CD1^+^C cells were purified by positive selection after depletion of PBMCs of CD19^+^B cells as previously described [34].

### The expression of IgE, IgG and mannose receptors by dendritic cells

The expression of FcϵRI, FcϵRII (CD23), FcγRI (CD64) and the mannose receptor was determined during the course of generating DCs, starting from day zero (monocyte) through to days 2, 4, 6 (immature) and 8 (mature). Receptor expression was also demonstrated on peripheral blood myeloid DCs (mDCs).

Following the isolation of monocytes from PBMCs and culturing them for DC generation, Mo-DCs (or mDCs were applicable) were harvested on each day of cell culture, washed with phosphate-buffered albumin (PBA; consisting of phosphate buffer saline, 0.5% bovine serum albumin and 0.1% sodium azide) and stained with anti-CD206 PC5, clone 19.2 (Pharmingen, San Diego, CA, USA), anti-CD23 ECD, clone 9P25, anti-CD64 (FcγRI) FITC, clone 22 (Beckman Coulter, High Wycombe, UK) and anti-FcϵRIα PE clone AER-37(CRA-1) (Cambridge Biosceince, Cambridge, UK) for 25 minutes in the dark at 4°C. The cells were then washed with PBA and fixed with 0.5% formaldehyde. Isotype control antibodies were used to determine binding specificity. Samples were analysed using flow cytometry as described before [42]. A minimum of 10,000 events were collected for each sample on a Beckman coulter FC500 flow cytometer. Data were analysed using WinMDI version 2.9. Cell populations of interest were initially gated on using forward and side scatter characteristics. For each sample the quadrants were typically set such that < 0.5% were positive in isotype control samples.

### IgE binding to Mo-DCs

Purified human IgE (Abbiotec, York, UK) was labelled with fluorescein using a Fluoro-Trap™ fluorescein labelling kit (Innova Biosciences Ltd, Cambridge, UK) according to the manufacturer’s instructions. The concentration of labelled IgE was determined by NanoDrop prior to use. In the assay for inhibition of labelled IgE uptake by DCs, unlabelled human IgE and IgG were used at different concentrations (Sigma-Aldrich, Irvine, UK) to stain 10^5^ immature Mo-DCs, which were first washed with PBA and tested as follows:

Three sets of DCs were prepared (10^5^ each). The first set was stained with IgE-FITC (7 μg/ml), the second set treated with unlabelled IgE (20 μg/ml) plus IgE-FITC (7 μg/ml) and the third set treated with IgG (20 μg/ml) plus IgE-FITC (7 μg/ml). Later, the cells were incubated for 20 minutes at 4°C and then washed with PBA and fixed. The detection of labelled IgE binding was performed using flow cytometry.

### IgE binding to Mo-DCs in the presence and absence of mannan

Four sets of DCs were prepared (10^5^ each). The first set was stained with IgE-FITC (7 μg/ml), the second set treated with 200 μg/ml mannan (MR ligand used as inhibitor) (Sigma-Aldrich, Irvine, UK) for 20 minutes at 37°C, washed and mixed with IgE-FITC (7 μg/ml), the third set incubated with Cy5-labeled Der p 1 for 20 minutes, while the fourth set was treated with 200 μg/ml mannan for 20 minutes at 37°C then washed and loaded with Cy5-Der p 1 for 20 minutes at 4°C followed by washing with PBA and fixing. The detection of labelled IgE and Der p 1 binding was performed using flow cytometry.

### Investigating the Der p 1 binding capacity of IgE and IgG1 2C7 antibodies by ELISA

The Der p 1 reactivity of IgE and IgG1 2C7 antibodies was investigated by ELISA. Maxisorp 96-well microtiter plates (Nunc, Roskilde, Denmark) were coated with Der p 1 (or deglycosylated Der p 1 (deg) where applicable) or the control allergen Fel d 1 (major allergen from the domestic cat *Felis domisticus*) (5 μg/ml) (Indoor Biotechnologies, Warminster, UK). The plates were incubated overnight at 4°C then washed three times using PBS-0.05% Tween-20 before blocking with PBS −1% BSA buffer for one hour at room temperature.

The coated plates were then washed and 2C7 antibodies, along with human IgE (Abbiotec, York, UK) and IgG (Sigma-Aldrich, Irvine, UK) control antibodies were incubated at different concentrations at 37°C for two hours. Subsequently, the plates were washed three times and the binding was detected by incubation with a goat anti-human κ chain HRP-conjugated secondary antibody (Sigma-Aldrich, Irvine, UK). After three washes with PBS-Tween, followed by a single wash with a proprietary HRP substrate buffer (24 mM citric acid and 52 mM Na2HPO4 in ddH20, pH 5.2), the plates were subsequently developed with o-Phenylenediamine (OPD) (Sigma-Aldrich, Irvine, UK) in a HRP substrate buffer containing 0.012% H_2_O_2_. The absorbance was measured at 492 nm.

### Testing the specificity of IgE-facilitated Der p 1 uptake by Mo-DCs

Mannan (200 μg/ml), MR’s natural ligand, was added to aliquots of Mo-DCs using uptake media containing RPMI (Sigma-Aldrich, Irvine, UK), 30% PBS with Ca^+2^ and Mg^+2^ (Gibco- Invitrogen, Paisley, UK) and 10% FBS (Autogen Bioclear, Wiltshire, UK). The cells were then incubated for 20 minutes at 37°C.

Different mixtures of Cy5-Der p 1 with or without human IgE or IgE 2C7 were prepared and incubated at 37°C for 20 minutes. These mixtures were added to cells that had either been pre-incubated with mannan or not, and then incubated for 20 minutes at 37°C. The final concentration of Der p 1 was 0.26 μg/ml, while human IgE and IgE 2C7 were used at 1 μg/ml. The cells were then washed and fixed with 0.5% formaldehyde.

In parallel, other cell aliquots were treated with 1 μg/ml or 10 μg/ml human IgE for 20 minutes at 4°C in order to block IgE receptors, following which they were washed and loaded with the mixture of Der p 1-IgE 2C7 as above for 20 minutes. The quantitative Cy5-Der p 1 uptake was then established using flow cytometry.

### Myeloid DC-T cell co-culture

Myeloid dendritic cells were incubated with Der p 1 or deg Der p 1 (0.26 μg/ml), with and without IgE 2C7, IgG 2C7, IgE or IgG (1 μg/ml), for two to three hours at 37˚C. Cells were then washed and cultured with autologous CD3 + CD45RO- (naïve) T cells at DC/T cell ratio of 1:5. The naïve T cells had been negatively separated from PBMCs, using a magnetic cell separation system (Miltenyi Biotec, Bisley, UK) and following the manufacturer’s instructions.

Cultures were carried out using 96-well U bottom plates (Nunc, Roskilde, Denmark) and RPMI 1640 supplemented with penicillin/streptomycin (Sigma-Aldrich, Irvine, UK) and 10% human AB serum (Sigma-Aldrich, Irvine, UK). IL-2 (20 IU, ml) (R & D system) was added every three to four days until day 10 of culture. After 10 days of culture, T cells were re-stimulated overnight with PMA (15 ng/ml) and Ionomycin (1 μg/ml). The supernatant from each condition was collected, and FlowCytomix Human Basic kits were used in order to detect cytokine concentrations.

### Statistical analysis

Student’s T test and Mann–Whitney test were used to perform the statistical analysis. Data was considered statistically significant when P-values were less than 0.05. *: p ≤ 0.05, ** p ≤ 0.01 and *** p ≤ 0.001.

## Competing interests

Authors have no competing interests.

## Authors’ contributions

IKS carried out the experimental work and drafted the manuscript. AA carried out deglycosylation studies. PF, KA and MK participated in generating the Der p 1-specific chimeric antibodies. FS, HS and AMG conceived of the study, and participated in its design and coordination and helped to draft the manuscript. All authors read and approved the final manuscript.
